# Toxic Metal Exposure from Ecuadorian Seafood: Dietary Exposure and Risk Characterization Across Key Commercial Species

**DOI:** 10.3390/foods15193428

**Published:** 2026-09-25

**Authors:** Wendy Daniela Carvajal, David Romero-Estévez, Gabriela S. Yánez-Jácome, Pamela Y. Vélez-Terreros, Hugo Navarrete

**Affiliations:** 1 Centro de Estudios Aplicados en Química (CESAQ), Pontificia Universidad Católica del Ecuador, Nayón 170530, Ecuador; wdcarvajal@puce.edu.ec (W.D.C.); dfromero@puce.edu.ec (D.R.-E.); gsyanez@puce.edu.ec (G.S.Y.-J.); pyvelez@puce.edu.ec (P.Y.V.-T.); 2 Facultad de Ciencias Exactas, Naturales y Ambientales, Pontificia Universidad Católica del Ecuador, Quito 170525, Ecuador

**Keywords:** cadmium, cobalt, lead, total mercury, shrimp-prawn, shrimp, finfish

## Abstract

Seafood is an important component of the Ecuadorian diet, particularly in coastal regions; however, contamination from anthropogenic activities has raised concerns about human exposure to toxic metals. This study quantified cadmium (Cd), cobalt (Co), lead (Pb), and total mercury (THg) in shrimp (*Protrachypene precipua*), shrimp-prawn (*Litopenaeus vannamei*), blue marlin (*Makaira nigricans*), and Pacific thread herring (PTH; *Opisthonema* sp.) purchased in Quito and Manta. A total of 71 samples were analyzed by atomic absorption spectrometry (flame and graphite furnace) and cold-vapor atomic fluorescence spectrometry. Distinct metal accumulation patterns were observed among species: blue marlin exhibited markedly elevated THg concentrations (1117.7 µg/kg) consistent with biomagnification processes, whereas shrimp-prawns showed the highest Cd levels (67.5 µg/kg). Pb and Co levels were below the corresponding limits of quantification in all samples. All detectable concentrations of Cd and THg remained below the maximum levels established by the Codex Alimentarius and European Union regulations. Risk characterization based on Hazard Quotient (HQ) indicated that THg intake from blue marlin exceeded the safety threshold (HQ > 1) across all demographic groups under both mean and maximum consumption scenarios. Additionally, HQ values above 1 were observed for toddlers and children consuming shrimp, shrimp-prawn, and PTH under the maximum exposure scenario. In contrast, Cd exposure remained below the level of concern for all seafood products and population groups. Overall, blue marlin was the primary contributor to potential mercury exposure, particularly among younger consumers.

## 1. Introduction

Seafood constitutes a fundamental component of the Ecuadorian diet, particularly in coastal regions, and it plays a strategic role in the national economy [1,2], with shrimp among the country’s most important export commodities and a major driver of the aquaculture sector [3,4,5]. This relevance largely stems from Ecuador’s extensive Pacific coastline and rich aquatic biodiversity, which support a wide variety of fish, crustaceans, and mollusks widely consumed as affordable sources of high-quality protein and essential micronutrients [6]. However, increasing pressure from anthropogenic activities such as mining, oil extraction, agriculture, and urban wastewater discharges has raised growing concerns about the chemical safety of seafood destined for human consumption [7,8,9].

Among chemical contaminants, toxic metals —including total mercury (THg), lead (Pb), and cadmium (Cd)—are of particular concern due to their persistence; their bioaccumulation, which can lead to diverse toxic effects across body tissues, organs, and ecosystems; and their potential for biomagnification along aquatic food webs [10,11,12,13,14,15,16,17]. These elements can enter marine and freshwater ecosystems through natural processes and, more significantly, through human activities, accumulating in sediments and aquatic organisms [16,18,19]. Several studies in Ecuador have documented elevated concentrations of toxic metals in commercially important fish and shellfish species, sometimes exceeding national and international regulatory limits [20,21].

Although the discussion on contamination in seafood usually focuses on highly toxic metals such as THg, Pb, and Cd, cobalt (Co) is also relevant because it is not only a trace element naturally present in the aquatic environment, but its concentration can also increase due to anthropogenic sources (mining, fossil fuels, sewage sludge, phosphate fertilizers, and industrial discharges), which enhances the likelihood of dietary exposure through seafood [22,23,24]. Furthermore, Co has a dual toxicological profile: on one hand, it is essential for humans as a component of vitamin B12 (cobalamin) [25]; on the other hand, excessive intake or prolonged exposure to Co can cause severe adverse effects affecting the cardiovascular, renal, hematological, and endocrine systems, and may also have carcinogenic potential depending on the specific compound and route of exposure [26,27,28,29].

Chronic exposure to Cd can cause bone demineralization and increase the risk of cancer [30,31,32]. Pb and THg (mainly methylmercury) have also been associated with cognitive deficits, neurodevelopmental disorders, and reduced IQ in children [10,33].

The public health implications of metal-contaminated seafood are especially relevant in populations with high consumption rates, including coastal and riverine communities [34]. Recent health risk assessments in Ecuador have identified potential non-carcinogenic risks associated with consuming certain fish species and bivalve mollusks, highlighting uneven exposure patterns across species, habitats, and trophic levels [35,36,37,38].

Despite the growing body of evidence, comprehensive assessments that integrate contamination levels and dietary exposure remain limited, particularly for marine seafood products frequently consumed in Ecuador. Moreover, studies on toxic metal occurrence in Ecuadorian shrimp and shrimp-prawn species remain scarce. Therefore, this study evaluates toxic metal exposure across several understudied seafood species, including shrimp and shrimp-prawns, as well as blue marlin and Pacific thread herring (PTH), as an initial approach to assess metal contamination and characterize consumer risk. These species are among the most widely consumed in coastal provinces and serve as key sources of animal protein, contributing substantially to national food security. Consequently, the present study examines the potential health risks associated with dietary toxic metal intake from their consumption.

## 2. Materials and Methods

### 2.1. Species Description

Ecuadorian seafood supply is anchored by several taxa spanning aquaculture and wild capture that differ markedly in trophic level, life history, and market pathways—differences directly relevant to toxic-metal bioaccumulation and human exposure. Among these species, the Pacific white shrimp (also known as whiteleg shrimp), *Litopenaeus vannamei* (syn. *Penaeus vannamei*), is native to the eastern tropical Pacific from Mexico to northern Peru [39], with adults occurring on coastal bottoms to depths of 72 m and juveniles recruiting to estuarine habitats [39,40,41]. In Ecuador, it has been cultivated since the 1970s and is now the sole farmed shrimp species [42,43], produced in earthen ponds (extensive to intensive) along the coast [44], with typical commercial yields near 7 t/(ha·yr) [45]. Globally, it is the most widely cultured shrimp, and Ecuador ranks among the leading producers and exporters, with 1.4 Mt exported in 2025, principally to China (49.45%), the European Union (21.68%), and the United States (19.41%) [46].

Another important fishery resource in Ecuador is the shrimp species *Protrachypene precipua* (locally known as “camarón pomada”). This species inhabits muddy bottoms within and outside estuary systems, as well as coastal marine areas of Ecuador, and generates an estimated annual revenue of US$ 4.5 million to US$ 5.0 million. Approximately 75% of the industrial sector’s production is exported primarily to the United States and the European Union [47,48].

For this study, *Litopenaeus vannamei* (Pacific white shrimp) is referred to as shrimp-prawn (reflecting its local Spanish name, *camarón-langostino*), whereas *Protrachypene precipua* is referred to simply as shrimp [39,47].

The blue marlin (*Makaira nigricans*, locally known as *“picudo”)* is a large, highly migratory, epipelagic species that inhabits tropical–subtropical oceanic waters [49] and attains lengths of up to 3.6 m and weights of up to 900 kg [50]. The blue marlin supports mixed commercial and recreational fisheries and is also taken as bycatch in industrial tuna fleets. As a long-lived, high-trophic-level predator with wide-ranging movements and market circulation (fresh/frozen), the blue marlin serves as a sentinel for biomagnified contaminants and for spatially heterogeneous exposure signatures across migratory routes, supporting risk appraisal in consumers [51,52,53,54].

The PTH (*Opisthonema* sp., locally known as “*pinchagua*” or “*machete de hebra*”) is a small, near-surface pelagic clupeoid (family Dorosomatidae) distributed throughout the eastern Pacific, from southern California and the Gulf of California to Peru, including Ecuador [55]. Ecuador hosts the most important regional fishery for this species [56], where PTH is harvested mainly by purse-seine fleets for direct human consumption (fresh or canned) and for fishmeal and fish oil production. Ecologically, it occupies a low trophic position as a plankton-feeding forage fish.

Collectively, these species occupy key positions in the aquatic food web (filter-feeding forage fish, farmed crustacean, apex teleost predator) and in supply chains (domestic consumption, export markets, and industrial ingredient production), providing a biologically and economically sound framework for assessing contamination, exposure, and risk of toxic metals in Ecuadorian seafood products.

### 2.2. Sampling and Metal Analysis

A total of 71 seafood samples were collected from retail markets and supermarkets in Quito, comprising 24 headless shrimps, 20 headless shrimp-prawns, and 16 blue marlin muscle samples. Additionally, 11 PTH samples were collected from open markets in Manta. Samples were purchased from various commercial vendors through multiple sampling campaigns between 2024 and 2026 to obtain a preliminary overview of metal occurrence in commercially available seafood. Information regarding production systems, capture locations, biological characteristics (such as size, age, and sex), and supply chain traceability was deemed unnecessary, as this study focuses primarily on seafood available for consumption. All samples were transported to the Centro de Estudios Aplicados en Química (CESAQ-PUCE) laboratory for further chemical analysis.

In the laboratory, each sample was rinsed with ≥1.0 MΩ·cm reagent-grade water, immediately frozen at −25 °C, and subsequently freeze-dried for 48 h (0.150 hPa, −50 °C) using a LaboGene CoolSafe system ( Allerød, Denmark). The percentage of water loss was recorded to convert analytical results from dry weight to wet weight. Dried tissues were then homogenized using a stainless-steel mill (Hamilton Beach, Glen Allen, VA, USA), with strict cross-contamination controls between samples, and stored in self-sealing plastic bags in a desiccator until acid digestion.

For metal determination, between 0.4 and 0.5 g (for Cd, Pb, and Co) and approximately 0.3 g (for THg) of homogenized material was weighed into high-pressure PTFE vessels (MARS EasyPrep, Matheus; NC, USA) and subjected to microwave-assisted acid digestion in a CEM MARS 6 (Matheus, NC, USA) system using 1 mL HNO_3_ (CAS# 7697-37-2), 1 mL H_2_O_2_ (CAS# 7722-84-1), and 1 mL HClO_4_ (CAS# 7601-90-3; all Fisher Chemical, Ottawa, ON, Canada, Certified ACS grade). Digested samples were subsequently diluted to a final volume of 25 mL. For THg analysis, 0.5 mL of concentrated HCl (Fisher Chemical, Ottawa, Canada, Certified ACS, CAS# 7647-01-0) was added to each sample before instrumental quantification. Cd and Pb were measured by graphite furnace atomic absorption spectrometry (GFAAS), and Co by flame atomic absorption spectrometry (FAAS), using a Pinnacle 900T THGA/FL/HSN, PerkinElmer, Inc. (Waltham, MA, USA). Total mercury was analyzed by cold vapor atomic fluorescence spectrometry (CVAFS; Analytik Jena Mercur Plus, Jena, Germany).

For all metals, multi-point external calibrations were prepared using NIST-traceable certified standards (Inorganic Ventures, Christiansburg, VI, USA). Analytical accuracy was verified in each digestion batch using the certified reference materials DORM-5 and SPIN-1 (National Research Council, Ottawa, ON, Canada) and by spiking real samples with known concentrations of each metal. Precision was evaluated using duplicate digestions within each digestion batch and instrumental replicates. All results were initially determined on a dry-weight basis and subsequently expressed on a wet-weight basis using the experimentally measured water content. The instrumental limit of detection (LOD) was determined based on the variability of ten digested blanks, calculated by multiplying the standard deviation of the blank concentration measurements by 3, while the limit of quantification (LOQ) was the lowest concentration of the linear range (Appendix A).

For exposure assessment and statistical analysis, left-censored data handling was applied for Cd and Hg concentrations below the LOQ; these values were set to one-half (½) of the respective LOQ for each metal [57,58], since only specific samples (Cd = 25% and Hg = 18%) fell below the limit. For Co and Pb, no data handling was applied because all samples fell below the LOQ, which would introduce significant bias to the mean and median values.

### 2.3. Exposure Assessment

To estimate human exposure to Cd and THg from seafood consumption, the Estimated Daily Intake (EDI) was calculated with Equation (1) [59] after a left-censored data approach to the below-LOQ values:

(1) $$EDI=\frac{C\times IR}{BW}$$

where C is the concentration of each metal (µg/kg, wet weight), IR is the ingestion rate (kg/week), and BW is the body weight (kg).

Body weight values were obtained from the Ecuadorian National Health and Nutrition Survey [60]. Mean BW of 12.2 kg for toddlers (<5 years), 27.8 kg for children (6–12 years), 53.0 kg for teenagers (13–18 years), and 67.9 kg for adults (>19 years) were used for calculations [60]. Ingestion rates (IR) were estimated using reference values from multiple sources. To align with the BW categories adopted from the Ecuadorian National Health and Nutrition Survey [60], the selected IR age ranges were matched closely with the corresponding age groups used for BW assignment in the exposure assessment. For adults (>19 years), an IR of 0.140 kg/day was adopted from the Ecuadorian Photographic Atlas of Food Portions [61]. Due to the lack of official Ecuadorian data on seafood consumption for toddlers, children, and teenagers, age-specific consumption rates were obtained from Chapter 10 (Intake of Fish and Shellfish, Table 10-9) of the U.S. EPA Child-Specific Exposure Factors Handbook [62]. This source provides age-specific estimates of daily fish and shellfish consumption. Accordingly, intake rates of 0.074 kg/day and 0.095 kg/day were applied to toddlers (<5 years) and children (6–12 years), respectively. For teenagers (13–18 years), an IR of 0.125 kg/day was used, representing the calculated mean of the intake values reported for the 11–15-year and 16–17-year age groups.

The Hazard Quotient (HQ) was calculated as the ratio between the estimated daily intake (EDI) and the respective oral reference dose (RfD) expressed in µg/kg day. The RfD values used were 1 for Cd and 0.1 for THg (considering the RfD for MeHg, as it is the most toxic organic form of Hg [58,59,63]. HQ values greater than 1 were interpreted as potentially concerning under the assumed intake scenarios.

According to the EPA 2000 [64], it is recommended to determine THg under the conservative assumption that all mercury in seafood tissues is present as MeHg. Therefore, analyzing THg serves as both a health-protective and cost-effective approach. In this study, we used the MeHg reference dose (RfD) and maximum permissible limit from international regulations for subsequent risk characterization assessments.

Because no data handling was performed for Co and Pb, exposure assessment and risk characterization were not conducted, as this could lead to over- or underestimation of the HQ.

### 2.4. Statistical Analysis

Statistical analyses were performed only for Cd and THg because Co and Pb concentrations were below the limit of quantification (LOQ) in all samples. The results were reported as the mean of replicates, where applicable. Coefficients of variation and recoveries of certified reference materials and spiked samples were calculated for quality assessment.

The non-parametric Kruskal–Wallis test was used to evaluate differences in Cd and THg concentrations among the four seafood products after applying a left-censored data approach. A pairwise Dunn test with Benjamini–Hochberg adjustment was used as a post hoc analysis to identify specific differences between seafood products. The false discovery rate (FDR) threshold was set at 0.05 as the *p*-value. The null hypothesis was rejected if and only if the adjusted *p*-value was less than or equal to FDR/2, as the stopping rule.

## 3. Results and Discussion

### 3.1. Quality Assessment of Results

Quality control evaluations indicated acceptable analytical performance for most target metals. For Cd-spiked samples, recoveries ranged from 72% to 123%, while certified reference materials showed recoveries of 95% to 107% for SPIN-1 and 97% for DORM-5. Co, THg, and Pb recoveries in spiked samples ranged from 84% to 114%, 80 to 94%, and 73 to 96%, respectively. CRM SPIN-1 Pb recoveries ranged from 76% to 125%.

### 3.2. Metal Content in Samples

Results for shrimp, shrimp-prawn, blue marlin, and PTH revealed a clear ecological distribution of toxic metals across species (Appendix B). Blue marlin stands out immediately for its exceptionally high Hg concentrations, with values exceeding or close to 1000 µg/kg in some samples (Figure 1). These elevated concentrations are fully consistent with their position as apex marine predators, where long lifespans and piscivorous feeding habits promote intense Hg biomagnification [38,65]. This phenomenon has been extensively documented for marlin, swordfish, and similar species, which consistently rank among the seafood products with the highest Hg burdens in regulatory assessments and risk evaluations [53,66,67]. Nevertheless, the highest Hg concentration detected in the present study (1117.7 µg/kg, assuming that the THg content measured is attributable to the methylmercury form) [64] remained below the maximum permissible limit for MeHg established by the Codex Alimentarius for marlin species: 1700 µg/kg [68].

In contrast, THg levels in PTH were moderate, generally ranging from 19.6 to 44.9 µg/kg (Figure 1), consistent with its lower trophic status. Mean THg levels in PTH were almost 22 times lower than in blue marlin. Small pelagic fish analogous to PTH, such as sardines and anchovies, typically accumulate far less THg than top predators do [69,70]. Shrimp samples showed the lowest THg concentrations overall (Figure 1), with values generally below 40.2 µg/kg. This observation is consistent with the established understanding that crustaceans tend to accumulate less MeHg than finfish because of their feeding ecology and shorter food-chain pathways [71,72]. This pattern mirrors U.S. Food and Drug Administration surveillance data, which show that species such as sardines and shrimp have among the lowest mercury levels [73]. THg content in shrimp-prawns was slightly higher than in shrimp (9.0 to 57.4 µg/kg), likely reflecting differences in lifespan, size, and cumulative bioaccumulation potential. THg in marine invertebrates is highly species-specific due to differences in ecological characteristics, such as feeding habits and habitats, and in the balance between uptake and excretion rates for each species [74,75]. Moreover, biological and ecological factors, including sex and seasonal variation, have been reported to significantly influence metal bioaccumulation, bioavailability, and, therefore, toxic metal transfer [76].

Mean THg concentrations followed the order: Blue marlin > PTH > shrimp-prawn > shrimp (Figure 1, Appendix C). The Kruskal–Wallis test revealed significant differences between THg levels among the seafood products (*p* < 0.05), indicating species-specific patterns of Hg accumulation. The post hoc comparisons (Dunn’s test with Benjamini–Hochberg adjustment) indicated statistically significant differences between shrimp-prawn and blue marlin (*Z* = 3.85, *p_adj_* < 0.025), shrimp and blue marlin (*Z* = 6.59, *p_adj_* < 0.025), shrimp and shrimp-prawn (*Z* = 2.76, *p_adj_* = < 0.025), and PTH and shrimp (*Z* = 3.68, *p_adj_* = < 0.025). Conversely, no significant differences were observed when comparing PTH with shrimp-prawn (*Z* = −1.35, *p_adj_* = 0.089) or blue marlin with PTH (*Z* = 2.01, *p_adj_* = 0.027).

In a study carried out by Garofalo et al. [77] on 5854 seafood samples from around the world, a Pacific marlin sample showed an extremely high Hg value of up to 13,000 µg/kg, exceeding the maximum limit of 1000 µg/kg set by the European Union [78], which is stricter than the Codex Alimentarius limit. However, in the same study, mean Hg levels in crustaceans were lower than in other taxonomic groups, such as marine fish, cephalopods, and bivalve mollusks, consistent with our results. Other studies have reported similarly low Hg levels in shrimps (up to 30 µg/kg), indicating that Hg concentrations do not depend on capture type (wild-caught or farm-raised) and that shrimps are generally considered a good choice for consumers [79].

Regarding Cd, mean concentrations were as follows: shrimp-prawn > blue marlin > shrimp > PTH (Figure 2, Appendix B). The Kruskal–Wallis test showed statistically significant differences (*p* < 0.05) among the seafood products studied. The post hoc analysis showed statistically significant differences between blue marlin and PTH (*Z* = 3.08, *p_adj_* < 0.025), shrimp and blue marlin (*Z* = 3.39, *p_adj_* < 0.025), PTH and shrimp-prawn (*Z* = 3.18, *p_adj_* = < 0.025), and shrimp and shrimp-prawn (*Z* = 3.57, *p_adj_* = < 0.025). In contrast, no significant differences were observed between blue marlin and shrimp-prawn (*Z* = 0.038, *p_adj_* = 0.4849) or between shrimp and PTH (*Z* = −0.31, *p_adj_* = 0.4519).

Shrimp-prawn samples predominated, with the highest Cd mean (19.5 µg/kg) and the highest single value in the dataset (67.5 µg/kg; Figure 2, Appendix B). Although this pattern is biologically expected (since crustaceans tend to concentrate Cd in the hepatopancreas), this organ was excluded from our analysis. It is generally not consumed and is removed with the head during shrimp and shrimp-prawn processing. However, Cd may still be present in other edible crustacean tissues at quantifiable levels, thereby contributing to human dietary exposure [80,81]. Although the Codex Alimentarius has not established maximum limits for Cd in the species analyzed here, all measured concentrations remained well below the 2000 µg/kg limit set for other marine organisms, such as bivalve mollusks and cephalopods [68], and below the 500 µg/kg maximum limit set for Cd in crustaceans by the European Union [78].

Studies on wild-caught shrimp and prawns repeatedly highlight Cd organotropism and the histopathological disturbances that prolonged exposure can produce in crustacean digestive tissues [80,82]. Ra et al. [83] reported high Cd concentrations in the intestine, whole body, and head, which differed significantly (*p* < 0.05) from the body, shell, and muscle, mainly due to the presence of the Cd-rich hepatopancreas in the shrimp head. High Cd bioaccumulation has been observed in the gills, followed by the hepatopancreas and muscles of prawns, with histopathological effects in these tissues under Cd exposure [84,85]. Other studies on prawns have also reported higher Cd levels, up to 2380 µg/kg [86], while Olowu et al. [87] reported mean Cd concentrations of 70 µg/kg in prawns from Nigeria, similar to the highest Cd levels observed in our study. In contrast, significantly higher Cd levels, reaching 7700 µg/kg, have been reported in shrimp from Bangladesh, indicating strong anthropogenic-driven bioaccumulation [88]. Conversely, other studies in the same region have detected much lower Cd concentrations. Sarkar et al. and Sultana et al. [89,90] reported levels up to 99 µg/kg, suggesting that Cd was not a major contaminant in those specific cases, even though lead, chromium, and arsenic could still pose potential consumer health risks. Similarly, Ahmed et al. [91] reported low Cd concentrations of 57–66 µg/kg in cultured shrimp from Bangladesh; however, this range is nearly twice our maximum observed Cd content. Furthermore, to the best of our knowledge, data regarding toxic metal levels in shrimp and prawns from Ecuador remain scarce.

The results of the present study revealed a mean Cd concentration of 15.8 µg/kg in blue marlin, with a maximum level of 28.4 µg/kg. Likewise, Cd content in PTH showed a similar pattern, peaking at 23.2 µg/kg. Notably, all Cd results in finfish remained well below the EU regulatory maximum of 50 µg/kg. In contrast, a study by Ruelas-Inzunza et al. [92] on blue marlin in the Gulf of California reported a minimum Cd accumulation level of 44 µg/kg, almost twice the levels observed in our study. Moreover, they found that other elements, including zinc, arsenic, and copper, exceeded Cd levels [92]. Similarly, other pelagic species have also shown low Cd levels. Ormaza-González et al. [93] found mean Cd levels of 30 µg/kg in tuna products from the eastern Pacific. According to Boalt et al. [94], several factors drive Cd distribution among fish species, including differences in uptake and elimination rates, trophic position, behavior, and habitat use. Interestingly, Cd accumulation tends to be higher in lower-trophic-level consumers than in higher-trophic-level consumers. To explain these interspecific variations in metal burdens, researchers emphasize intrinsic biotic factors, such as differing physiological growth rates or elevated uptake from high feeding rates [94].

Unlike THg and Cd, Pb was not detected in any samples, with all values below the LOQ (55.8 µg/kg w.w.). Consequently, no species-specific accumulation pattern could be identified. These results should be interpreted as concentrations below the method’s analytical quantification limit, rather than as evidence of negligible contamination or compliance with lower regulatory thresholds. Similar findings of non-quantifiable or low Pb levels have also been documented in shrimp [83,95], prawns [87], and finfish [77].

Cobalt concentrations were also below the LOQ (558.1 µg/kg w.w.) in all analyzed samples. However, our results do not exclude its presence at concentrations below the LOQ. No evidence of species-dependent cobalt accumulation was observed, despite the broad variability in Co concentrations reported in the literature for marine organisms [24,96,97]. Furthermore, although the Codex Alimentarius has not established maximum limits for Co in foods, the lack of quantifiable concentrations across all species analyzed indicates a generally low trace-metal burden in these products.

Across species, internal variability was evident. Blue marlin, for example, exhibited Hg concentrations ranging from below the LOQ to 1117.7 µg/kg (Figure 1), likely reflecting differences in age, feeding behavior, migratory routes, and capture location. Such variability among large pelagic predators is well documented and frequently linked to ecological niche and prey composition [65,98]. Likewise, the higher Cd values observed in certain shrimp-prawn samples may reflect localized environmental conditions and differences in size-related accumulation.

Taken together, the dataset revealed a coherent ecological pattern: blue marlin accumulated THg at substantially higher levels than the other seafood products, whereas shrimp-prawns showed a greater tendency to retain Cd through physiological accumulation mechanisms. From both public health and environmental monitoring perspectives, this first approach to toxic metal contamination in species with different ecological distributions provides reassuring results and strong evidence of a favorable contamination profile for the analyzed seafood products.

### 3.3. Human Health Exposure Assessment

Estimated daily intake (EDI) and Hazard Quotient (HQ) for Cd and THg in the four seafood products under study were evaluated (Table 1).

The calculated EDIs for Cd in shrimp, prawns, blue marlin, and PTH were below the RfD. Therefore, the HQ values were consistently well below 1 across all species and demographic groups (Table 1). The highest observed value was 0.410, found in the maximum-intake scenario for toddlers consuming shrimp-prawns. Other studies have reported similar findings regarding Cd exposure through the consumption of shrimp, shrimp-prawns, and fish, concluding that current exposure levels remain below toxicological concern for both children and adults [99,100,101,102,103]. These findings also align with global exposure assessments indicating that cereals, vegetables, and tubers—not seafood—are the primary drivers of dietary Cd intake in the general population [104,105].

Nevertheless, because of cadmium’s long biological half-life, the Joint FAO/WHO Expert Committee on Food Additives (JECFA) established a monthly tolerable intake of 25 µg/kg BW [31], rather than a weekly or daily value. Exceeding this JECFA threshold through heavy seafood consumption can increase the risk of chronic illnesses, including renal failure [91]. Therefore, continuous monitoring of toxic metal contamination in seafood is essential to protect consumers and keep exposure within acceptable toxicological limits [106].

Total mercury exposure showed markedly different patterns among the evaluated seafood species. Blue marlin consistently presented the highest THg exposure estimates across all age groups and consumption scenarios. Under the mean exposure scenario, THg HQ values ranged from 14.12 in adults to 41.54 in toddlers, substantially exceeding the threshold of 1. Meanwhile, under the maximum THg concentration (worst-case scenario), HQ values increased up to 67.79 in toddlers, 38.19 in children, 26.36 in teenagers, and 23.05 in adults, indicating a potential non-carcinogenic risk associated with blue marlin consumption. These findings indicate that blue marlin is consistently associated with a potential risk from Hg exposure. This result aligns with international evaluations indicating that high consumers of large predatory fish, particularly women of reproductive age and children, may exceed health-based thresholds due to Hg biomagnification [78,107,108]. In contrast, considering maximum scenarios, the HQ values exceeding the safety threshold were observed for shrimp in toddlers and children (HQ = 2.44 and 1.37, respectively), for shrimp-prawns in all age groups (HQ = 1.18–3.48), and for PTH in toddlers (HQ = 2.72), children (HQ = 1.53), and teenagers (HQ = 1.06).

According to a study by Médieu et al. [109] on four billfish species from the western Indian and southwestern Pacific Oceans, blue and black marlin are generally considered safer for consumption than swordfish and striped marlin with respect to MeHg exposure. However, consumption frequency should decrease as fish size increases, because Hg bioaccumulates with body size and age. Accordingly, health authorities recommend that pregnant women and young children limit or avoid the consumption of large pelagic predatory fish [37,109]. Furthermore, because fish and seafood are key components of the human diet, particularly in coastal regions of Ecuador and many other areas worldwide, it is crucial to raise public awareness of the potential health risks of mercury exposure through seafood consumption [110].

Cobalt and Pb concentrations were below their respective LOQs in all analyzed samples; therefore, EDI and HQ values were not calculated. Consequently, the available data did not support a quantitative dietary exposure assessment. Nevertheless, the lack of quantifiable concentrations across all evaluated seafood species indicates that any dietary contribution from these metals lies below the analytical sensitivity achieved in this study. Future investigations utilizing lower limits of quantification or larger sample sizes could better characterize background exposure levels and support a more comprehensive health risk assessment.

The relative susceptibility observed among age groups aligns with well-established toxicokinetic principles of toxic metal absorption and distribution. Toddlers exhibited the highest EDI and HQ values, followed by children, teenagers, and adults. Several studies have demonstrated that children are more vulnerable to toxic metal exposure than adults because of their higher metabolic and physiological activity and immature detoxification systems, among other factors, which result in greater absorption of toxic metals relative to body weight [111,112,113,114,115]. Toxicological profiles from the ATSDR [22,30,33,116] emphasize that body-weight–normalized intake and developmental status critically modulate toxicokinetic behavior and vulnerability, particularly to Hg compounds. Moreover, extensive biomonitoring evidence shows that children are more susceptible to metals such as Hg because of immature physiological systems, distinct exposure pathways, and greater gastrointestinal absorption efficiency than adults, reinforcing the importance of age-stratified risk assessment [117,118]. Our findings support implementing age-specific fish consumption advisories, especially for high-Hg species such as blue marlin, which remains a primary source of dietary MeHg exposure. Additionally, continuous monitoring and control are essential to prevent the consumption of foods with elevated levels of toxic metals.

## 4. Conclusions

The present study found the highest THg levels in predatory fish (blue marlin), while PTH, shrimps, and shrimp-prawns showed lower THg and Cd levels. These findings align with the maximum permissible limits established by FAO and EU regulations. Our results indicate that THg exposure from blue marlin poses a consistent concern across all demographic groups under both typical and high-consumption assumptions. Consequently, limiting the consumption of large pelagic predators is recommended. In contrast, shrimp, prawn, and PTH pose minimal THg risk. Cd exposures were consistently well below conservative health-based limits across all evaluated scenarios.

Since Pb and Co concentrations were below the limit of quantification across all analyzed samples, their respective EDI and HQ values could not be calculated. Nevertheless, the absence of quantifiable concentrations suggests that dietary exposure from the analyzed seafood products is likely low, relative to the analytical sensitivity achieved in this study. Moreover, certain limitations emerged, as the EDI and subsequent HQ calculations were constrained by the lack of up-to-date national data on seafood consumption rates across Ecuadorian regions, where consumption patterns vary significantly.

Furthermore, studies on toxic metal occurrence in Ecuadorian shrimp and shrimp-prawn species remain scarce; thus, this study provides a novel approach to address this critical knowledge gap. Although existing research highlights the presence of toxic metals in widely consumed fish species, further studies of other taxonomic groups, such as crustaceans, are crucial, given their relatively higher Cd levels than fish.

## Figures and Tables

**Figure 1:**
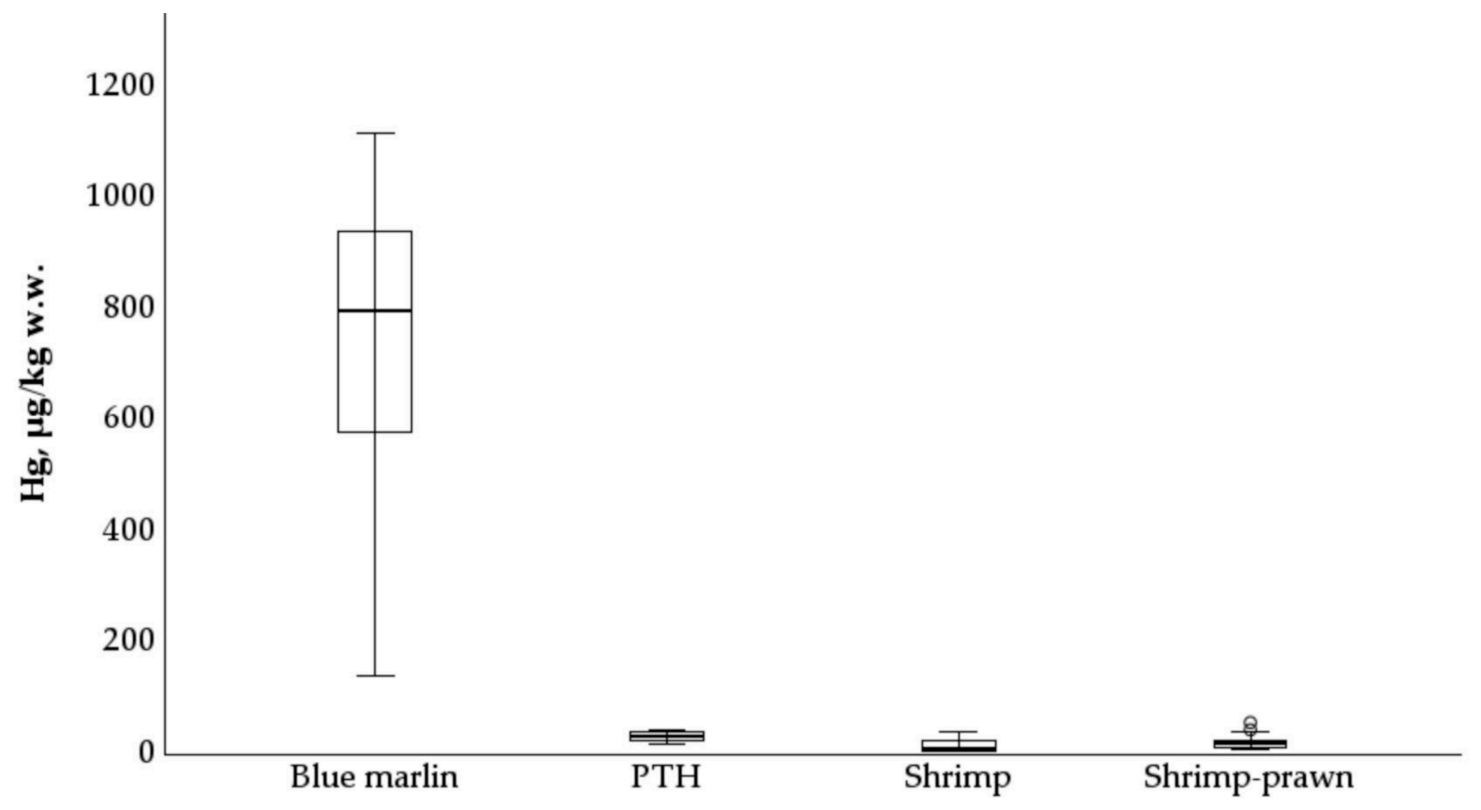
Total mercury content (µg/kg) expressed in wet weight (w.w.) of the analyzed seafood products.

**Figure 2:**
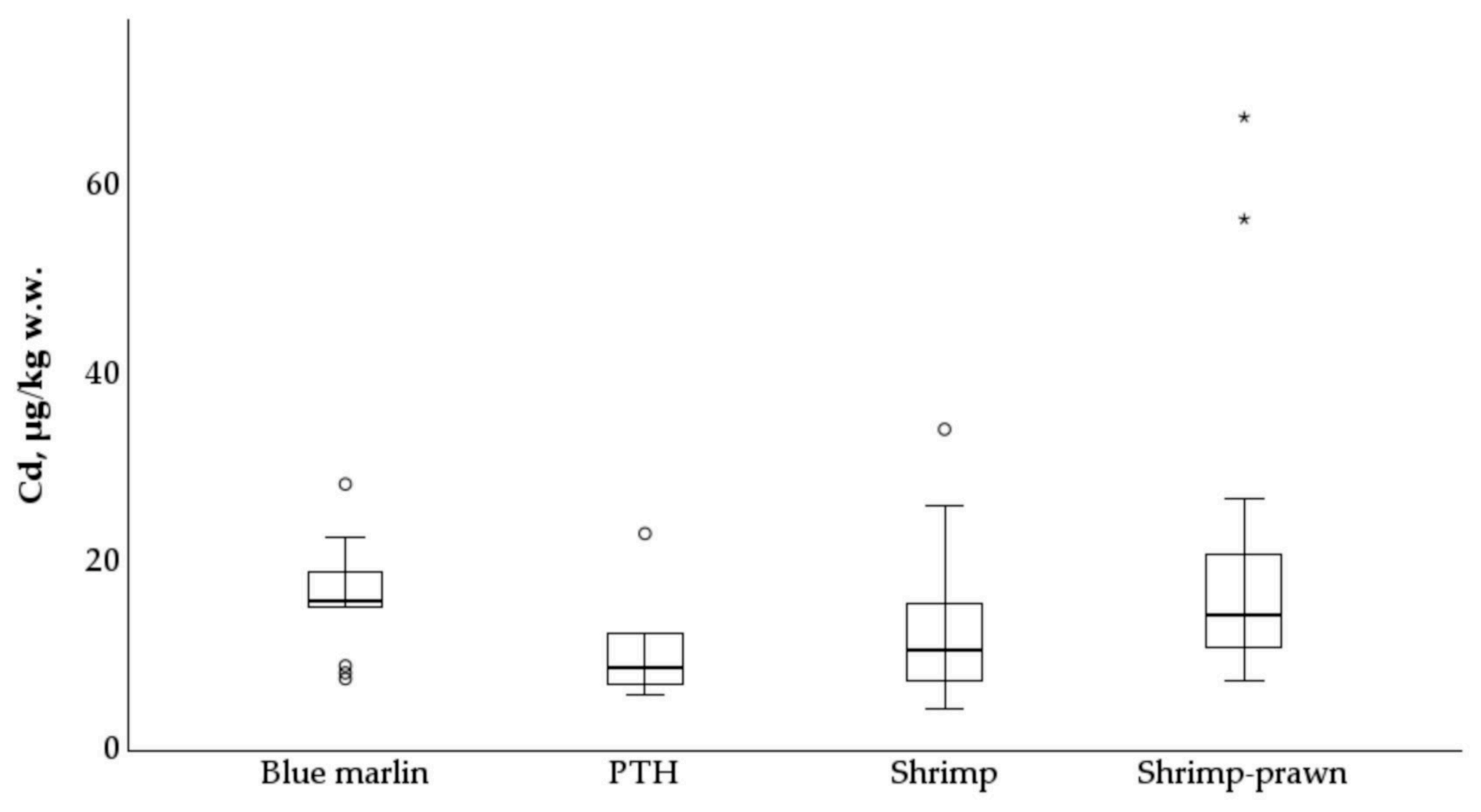
Cadmium content (µg/kg) expressed in wet weight (w.w.) of the analyzed seafood products. The symbol (*) represents the values located more than three times the interquartile range above the third quartile.

**Table 1:** Toxic metal concentrations (Cd, Hg) in shrimp, shrimp-prawn, blue marlin, and Pacific thread herring samples, including mean, minimum, and maximum values, along with estimated daily intake (EDI) and hazard quotient (HQ) across four age groups.

Metal			Cadmium			Mercury		
Samples	Values		Mean	Min	Max	Mean	Min	Max
Shrimp (*n* = 24)	Concentration (µg/kg)		8.7	1.7	34.3	10.1	2.7	40.2
	EDI (µg/kg BW day)	Toddlers (<5 years)	0.053	0.010	0.208	0.061	0.017	0.244
		Children (6–12 years)	0.030	0.006	0.117	0.034	0.009	0.137
		Teenagers (13–18 years)	0.021	0.004	0.081	0.024	0.006	0.095
		Adults (>19 years)	0.018	0.003	0.071	0.021	0.006	0.083
	HQ	Toddlers (<5 years)	0.053	0.010	0.208	0.611	0.166	2.438
		Children (6–12 years)	0.030	0.006	0.117	0.344	0.093	1.373
		Teenagers (13–18 years)	0.021	0.004	0.081	0.238	0.064	0.948
		Adults (>19 years)	0.018	0.003	0.071	0.208	0.056	0.829
Shrimp-prawn (*n* = 20)	Concentration (µg/kg)		19.5	7.4	67.5	21.8	9.0	57.4
	EDI (µg/kg BW day)	Toddlers (<5 years)	0.119	0.045	0.410	0.132	0.055	0.348
		Children (6–12 years)	0.067	0.025	0.231	0.075	0.031	0.196
		Teenagers (13–18 years)	0.046	0.017	0.159	0.051	0.021	0.135
		Adults (>19 years)	0.040	0.015	0.139	0.045	0.019	0.118
	HQ	Toddlers (<5 years)	0.0119	0.045	0.410	1.322	0.549	3.479
		Children (6–12 years)	0.067	0.025	0.231	0.745	0.309	1.960
		Teenagers (13–18 years)	0.046	0.017	0.0159	0.514	0.213	1.353
		Adults (>19 years)	0.040	0.015	0.139	0.450	0.186	1.183
Blue marlin (*n* = 16)	Concentration (µg/kg)		15.8	4.6	28.4	684.9	6.0	1117.7
	EDI (µg/kg BW day))	Toddlers (<5 years)	0.096	0.028	0.172	4.154	0.037	6.779
		Children (6–12 years)	0.054	0.016	0.097	2.340	0.021	3.819
		Teenagers (13–18 years)	0.037	0.011	0.067	1.615	0.014	2.636
		Adults (>19 years)	0.033	0.009	0.059	1.412	0.012	2.304
	HQ	Toddlers (<5 years)	0.096	0.028	0.172	41.541	0.366	67.793
		Children (6–12 years)	0.054	0.016	0.097	23.404	0.206	38.193
		Teenagers (13–18 years)	0.037	0.011	0.067	16.153	0.142	26.360
		Adults (>19 years)	0.033	0.009	0.059	14.121	0.124	23.045
PTH (*n* = 11)	Concentration (µg/kg)		7.0	3.7	23.2	32.3	19.6	44.9
	EDI (µg/kg BW day)	Toddlers (<5 years)	0.042	0.023	0.141	0.196	0.119	0.272
		Children (6–12 years)	0.024	0.013	0.079	0.110	0.067	0.153
		Teenagers (13–18 years)	0.017	0.009	0.055	0.076	0.046	0.106
		Adults (>19 years)	0.014	0.008	0.048	0.067	0.040	0.093
	HQ	Toddlers (0–5 years)	0.042	0.023	0.141	1.959	1.186	2.721
		Children (6–12 years)	0.024	0.013	0.079	1.103	0.668	1.533
		Teenagers (13–18 years)	0.017	0.009	0.055	0.762	0.461	1.058
		Adults (>19 years)	0.014	0.008	0.048	0.666	0.403	0.925

**Figure A1:**
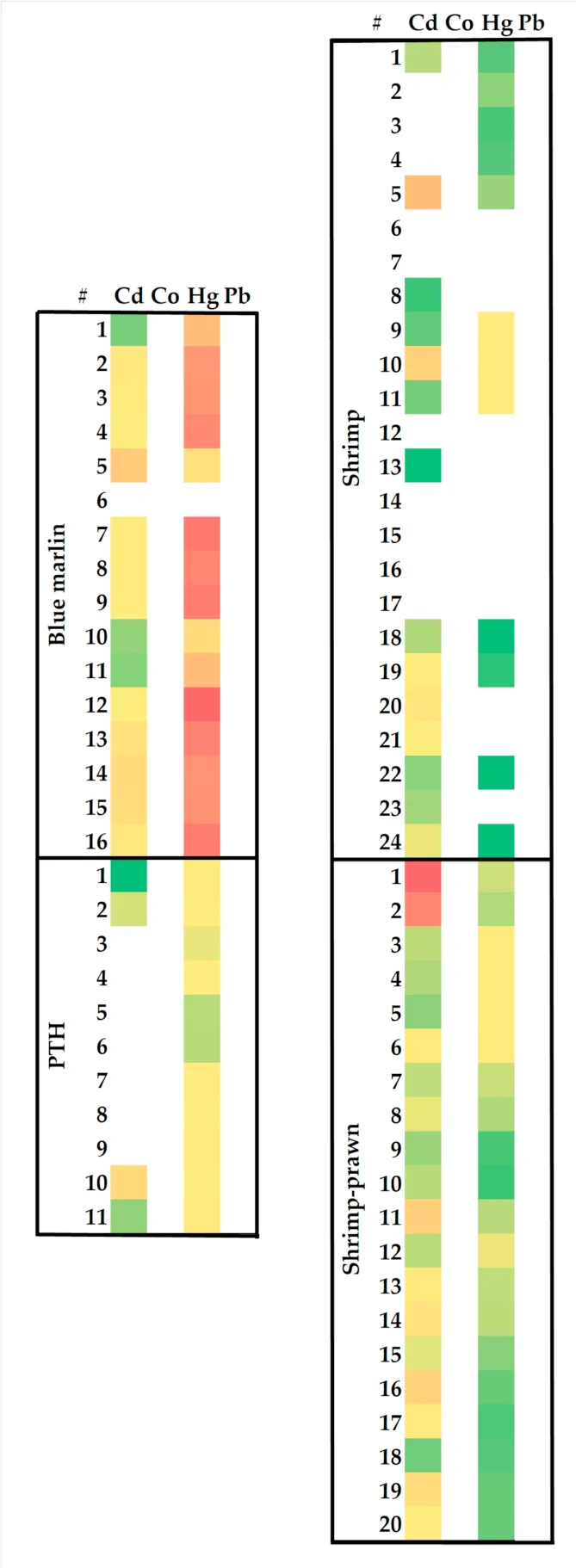
Species-specific distribution patterns of cadmium (Cd), cobalt (Co), mercury (Hg), and lead (Pb) in shrimp, prawn, blue marlin, and Pacific thread herring (PTH) samples analyzed in this study. Color scale: green = lowest concentration; yellow = intermediate concentration; red = highest concentration.

## Data Availability

The original contributions presented in this study are included in the article. Further inquiries can be directed to the corresponding author.

## Abbreviations

The following abbreviations are used in this manuscript:

PTH	Pacific thread herring
CESAQ-PUCE	Centro de Estudios Aplicados en Química de la Pontificia Universidad Católica del Ecuador
PTFE	Polytetrafluoroethylene
THg	Total mercury
FAAS	Flame atomic absorption spectrometry
GFAAS	Graphite furnace atomic absorption spectrometry
CVAFS	Cold vapor atomic fluorescence spectrometry
EWI	Estimated weekly intake
HQ	Hazard quotient
PTWI	Provisional tolerable weekly intake

## Appendix A

**Table A1:** Detection limits, quantification limits, and calibration parameters of the analytical methods used for trace metal determination.

Parameter	Cadmium (GFAAS)	Cobalt (FAAS)	Lead (GFAAS)	Mercury (CVAFS)
Limit of Detection * (µg/kg w.w.)	1.1	137.1	9.1	1.1
Limit of Quantification ** (µg/kg w.w.)	3.8	558.1	55.8	8.7
Instrumental linear range (µg/L)	0.5–4.0	50–1500	5.0–40.0	0.5–5.0
Coefficient of determination *R*^2^	>0.999	>0.999	>0.999	>0.999

Note: GFAAS: graphite furnace atomic absorption spectrophotometry; FAAS: flame atomic absorption spectroscopy; CVAFS: cold vapor atomic fluorescence spectrometry; w.w.: wet weight (fresh weight). * Experimentally determined, expressed in wet weight. ** Lowest concentration of the instrumental linear range expressed in wet weight.

## Appendix C

**Table A2:** Descriptive statistics of quantifiable cadmium and total mercury concentrations (µg/kg) in the analyzed seafood products.

Metal	Species	n	Concentration Range of Results	Median	Mean	SD
Cadmium	Shrimp	24	4.4—34.3	10.6	13.4	8.3
	Shrimp-prawn	20	7.4—67.5	14.5	19.5	15.6
	PTH	11	7.0—23.2	10.7	12.9	7.2
	Blue marlin	16	7.7—28.4	16.0	16.6	7.7
Mercury	Shrimp	24	5.8—40.2	10.5	16.7	13.0
	Shrimp-prawn	20	9.0—57.4	19.0	21.8	13.4
	PTH	11	19.5—44.9	32.7	32.3	8.89
	Blue marlin	16	141.6—1117.7	797.3	730.1	300.0

**Table A3:** Descriptive statistics of cadmium and total mercury concentrations (µg/kg) in the analyzed seafood products. Left-censored data (concentrations below the quantifiable limit, LOQ), were handled by assigning a value of one-half of the respective LOQ.

Metal	Species	n	Concentration Range of Results	Median	Mean	SD
Cadmium	Shrimp	24	1.7—34.3	6.2	8.7	8.4
	Shrimp-prawn	20	7.4—67.5	14.5	19.5	15.6
	PTH	11	7.0—23.2	4.2	7.0	6.0
	Blue marlin	16	4.6—28.4	15.9	15.8	6.2
Mercury	Shrimp	24	2.7—40.2	5.0	10.1	11.2
	Shrimp-prawn	20	9.0—57.4	19.0	21.8	13.4
	PTH	11	19.5—44.9	32.7	32.3	8.9
	Blue marlin	16	6.0—1117.7	786.4	684.9	341.7
